# Generation to Generation Project: Pairing Students With Older Adult Mentors During the COVID-19 Pandemic

**DOI:** 10.1093/geroni/igab046.294

**Published:** 2021-12-17

**Authors:** Pamela Elfenbein

**Affiliations:** University of North Georgia, Gainesville, Georgia, United States

## Abstract

To meet the needs of older adults isolated in the midst of the COVID-19 pandemic, we began pairing Human Services and Gerontology students with community dwelling adults 55 years of age and older, recruited through senior centers and other organizations serving older adults.

Students paired with isolated older adults were tasked with building supportive relationships, assuring that basic needs were being met and that all available community resources were in place, and then documenting their mentor’s life stories with an emphasis on the resilience, strength and wisdom of their older adult mentors (to focus and build on their strengths, the older adults we engage with are referred to as our “mentors”). Oral histories and supporting artifacts are archived in the university’s library permanent repository.

The oral histories portray unique perspectives into life, strength, and resilience during the COVID-19 pandemic and quarantine. The older adult mentors participating in the Generation to Generation project reported feeling strengthened and connected through their participation in the Generation to Generation project.

To determine if social Isolation and loneliness can be ameliorated through participation in the Generation to Generation oral history project, researchers utilized the UCLA Loneliness Scale in pre- and post- participation interviews; findings will be shared.

